# Seed shattering: from models to crops

**DOI:** 10.3389/fpls.2015.00476

**Published:** 2015-06-24

**Authors:** Yang Dong, Yin-Zheng Wang

**Affiliations:** State Key Laboratory of Systematic and Evolutionary Botany, Institute of Botany, Chinese Academy of Sciences, Beijing, China

**Keywords:** seed shattering, fruit shedding, pod dehiscence, domestication, domestication syndrome, indehiscent fruit, genetic regulation

## Abstract

Seed shattering (or pod dehiscence, or fruit shedding) is essential for the propagation of their offspring in wild plants but is a major cause of yield loss in crops. In the dicot model species, *Arabidopsis thaliana*, pod dehiscence necessitates a development of the abscission zones along the pod valve margins. In monocots, such as cereals, an abscission layer in the pedicle is required for the seed shattering process. In the past decade, great advances have been made in characterizing the genetic contributors that are involved in the complex regulatory network in the establishment of abscission cell identity. We summarize the recent burgeoning progress in the field of genetic regulation of pod dehiscence and fruit shedding, focusing mainly on the model species *A. thaliana* with its close relatives and the fleshy fruit species tomato, as well as the genetic basis responsible for the parallel loss of seed shattering in domesticated crops. This review shows how these individual genes are co-opted in the developmental process of the tissues that guarantee seed shattering. Research into the genetic mechanism underlying seed shattering provides a premier prerequisite for the future breeding program for harvest in crops.

## Introduction

The emergence of fruit represents a major evolutionary innovation in angiosperms, and the evolutionary success of wild plant species depends essentially on their capacity to scatter their offspring (Nathan and Muller-Landau, 2000). The seed shattering or fruit shedding is usually used to describe the detachment of the fruit from the pedicel in cereals and fleshy fruit species, respectively. While in dry dehiscent fruit taxa, such as Legumes and crucifers, pod dehiscence refers to the shattering of the pod shell, which enable the successful shattering of seeds. Although these processes happen in non-homologous tissues, the abscission layer is an essential tissue both for the shattering or shedding process (Estornell et al., 2013).

The fruit morphology and associated dispersal strategies are of significant adaptive importance, which are under strong selective pressures. While in seed crops, premature seed shattering is an undesired character and has been selected against during the domestication process of distinct crops. Most of our knowledge on the genetic regulation of pod dehiscence has been obtained in the model organism *Arabidopsis thaliana*, a Brassicaceae species with a characteristic dry dehiscent fruit that shatters the seeds through the dehiscence zones (DZs) along the silique after maturity (Ferrándiz et al., 1999). The differentiation of the DZ is under the control of intricate regulatory networks involving multiple transcription factors. Recent investigations in pod dehiscence regulation have uncovered another layer of the regulatory network that include phytohormones in specifying the DZs (Sorefan et al., 2009; Arnaud et al., 2010; Marsch-Martinez et al., 2012). Evidence from comparative studies in the taxa related to *Arabidopsis* suggests modest genetic changes in the key regulatory component could be responsible for the phenotypic changes that are associated with fruit function and novel dispersal strategies (Avino et al., 2012; Fourquin et al., 2013; Mühlhausen et al., 2013). Studies on the fruit shedding process in tomato, a model for fleshy fruits, have provided new insights into the regulatory networks responsible for the control of cell separation (Mao et al., 2000; Nakano et al., 2012; Liu et al., 2014). These findings reveal that there are strong similarities between dry and fleshy fruits in the molecular networks governing fruit dehiscence and maturation. Meanwhile, our understanding about the genes involved in the loss of seed shattering in crops has increased dramatically, offering us a great opportunity to examine the details regarding the molecular basis of such convergent morphological adaptation in the face of artificial selection in a wide array of species.

In this review, we try to incorporate the recent insights into the molecular and hormonal regulation of tissues that are necessary for seed shattering and fruit shedding in model species and discuss how the genetic modification of the regulatory genes is co-opted in the evolutionary process to generate altered fruit morphologies with novel dispersal strategies. We also review the recent findings in the genetic control of non-shattering (indehiscent) fruit in crop species and highlight the prevalence of parallel molecular evolution in plant domestication. A comprehensive understanding of the factors influencing the seed shattering process is particularly important, as it might have great potential in the facilitation of future crop domestication and breeding procedures to prevent unwanted seed loss.

## Genetics of Pod Dehiscence in *Arabidopsis thaliana* and its Relatives

**FIGURE 1:**
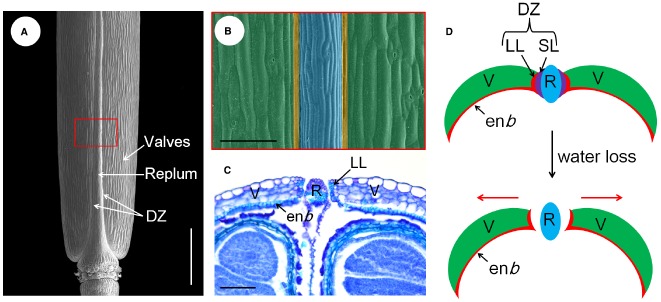
**Tissue organization and pod dehiscence process of the *Arabidopsis* fruit. (A)** Scanning electron microscopic (SEM) micrograph of a mature silique, the different parts are indicated. **(B)** A close-up view of the red boxed area shown in **(A)**, the valve, DZ, and replum are shaded with green, yellow, and blue color, respectively. **(C)** transversal section of the ovary region of a mature silique showing the SL has already been disintegrated and the silique opens from the replum. **(D)** Models for the pod dehiscence process of *Arabidopsis*, not to scale. The red arrows indicate the mechanical force generated in the valves. DZ, dehiscence zone; en*b*, endocarp *b* layer; LL, lignified layer; R, replum; SL, separation layer; V, valves. Scale bars in **(A)**, 1.5 mm; **(B,C)**, 80 μm.

The model species *A. thaliana* belongs to the Brassicaceae family, which develops a typical dry dehiscent fruit called the silique. Essentially, the silique develops from the gynoecium composed of two congenitally fused carpels (Ferrándiz et al., 1999). The developmental program of the fruit initiates from fertilization of the ovules. In the transverse view of the mature fruit, the out layer consists of three principal tissues, the valves, the replum, and the valve margins. The valve margins are sandwiched between the valve and replum and are further differentiated into lignified layer (LL) and separation layer (SL), which together form the DZ along the silique (Figures 1A–C; Ferrándiz et al., 1999). The LL cells are connected with the endocarp *b* (en*b*) layer of the valves, which is also rigidly lignified. The SL is composed of several isodiametric cells, and will be degraded autonomously before pod dehiscence (Seymour et al., 2013). When the silique becomes dry with loss of water, these highly organized structures produce a spring-like tension within the pod valves that force the silique to shatter from the weakest position, the SL (Figures 1C,D). Therefore, the silique dehiscence is a dynamic process that depends on the proper positioning and formation of the DZs along the silique (Ferrándiz, 2002).

### The Genetics of DZ Development and Pod Dehiscence in *Arabidopsis*

**FIGURE 2:**
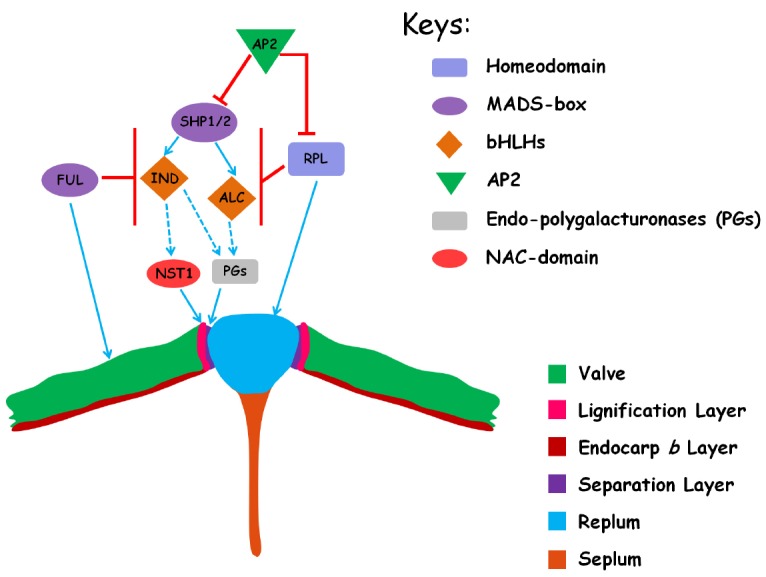
**The regulatory network for the differentiation of tissues that are necessary for pod dehiscence in *Arabidopsis*.** The cartoon represents a transversal section in the mature ovary and only the replum region is shown. The different regulatory genes and tissues are indicated by distinct color in the right. The positive relationships between genes by direct evidence are shown by blue arrows and the indirect relationships are shown in dashed arrows. The negative relationships between genes are shown in red bars.

The spatial specification of DZ, valve cells and replum is under the control of a complex genetic regulatory network and dynamic hormonal interactions with several transcription factors involved (Figure 2; Lewis et al., 2006; Østergaard, 2009; Ferrándiz and Fourquin, 2014). This regulatory network has recently been extended to include genes that are involved in the leaf development and the establishment of dorsoventral axes of the lateral organs (e.g., *FILAMENTOUS FLOWER*, *YABBY3*, *ASYMMETRIC LEAVES1/2*) as well as the meristematic potential maintenance (*BREVIPEDICELLUS*) (Hay et al., 2006; Alonso-Cantabrana et al., 2007). This review mainly focuses on the core regulatory genes specific to silique dehiscence, thus those remotely related genes are not included in this article. A thorough description of all these interactions can be found elsewhere in the literatures (Dinneny et al., 2005; Lewis et al., 2006; Østergaard, 2009).

Two MADS-box transcription factor encoding genes *SHATTERPROOF1* (*SHP1*) and *SHP2* act redundantly to control the pod dehiscence as neither single mutant displays a detectable phenotype from wild type (Liljegren et al., 2000). The *shp1/2* double mutant produces indehiscent fruit devoid of cell differentiation in the DZ (Liljegren et al., 2000). Expressions of *SHP1/2* are specifically localized in the DZs and developing seeds during late fruit development (Liljegren et al., 2000; Colombo et al., 2009). Further genetic analysis shows that *SHP1/2* act at the top of the genetic cascade that direct the development of DZ for pod dehiscence (Figure 2; see below; Ferrándiz, 2002; Lewis et al., 2006).

Acting down-stream of and in parallel with *SHP1/2* are two b-HLH transcription factors, *INDEHISCENT* (*IND*) and *ALCATRAZ* (*ALC*; Figure 2). *IND* directs the differentiation of DZ into LLs and SLs. Similar to *shp1/2* double mutant, *ind* mutation fully abolishes the specification of DZs and results in indehiscent fruits (Liljegren et al., 2000, 2004). By contrast, *ALC* specifically establishes the cell identity in the separation layer and mutation in *ALC* leads to partially indehiscent fruits (Rajani and Sundaresan, 2001). Both *IND* and *ALC* are specifically expressed in the DZ during late fruit development. Evidence indicates that *IND* acts downstream of *SHP1/2* to control pod dehiscence, as illustrated by the observation that *IND* expression is completely lost in the *shp1/2* mutant (Liljegren et al., 2000, 2004; Rajani and Sundaresan, 2001). The valve identity is regulated by the activity of the *FRUITFULL* (*FUL*) MADS-box gene (Gu et al., 1998; Ferrándiz et al., 2000). Expression of *FUL* initiates in the carpel primordia very early in flower development, and soon after becomes restricted in the gynoecium and further in the carpel valves (Gu et al., 1998). In the *ful* mutant, the valves fail to elongate and are cracked by the inner developing seeds (Gu et al., 1998). *FUL* negatively regulates *SHP1/2* expression thus delimitates the boundary of *SHP1/2* expression in the valves (Ferrándiz et al., 2000). When *FUL* is mutated, *SHP1/2* and *IND* are ectopically expressed in the valves promoting the mesocarp cells to adopt lignified valve margin cell identity instead of normal parenchymatous cell identity (Gu et al., 1998; Ferrándiz et al., 2000). The *ful* mutant phenotype can be partially rescued by combining the *ful* mutant with mutations in the *SHP1/2* genes, and largely rescued in the *ind* mutant background, suggesting that *IND* has a more specialized role in DZ cell specification than *SHP1/2*. On the other hand, fruits of *35S::FUL* transgenetic lines are indehiscent as the result of complete conversion of DZ cells into valve cells (Ferrándiz et al., 2000). Interestingly, the activity of *SHP1/2*, *IND*, *ALC*, and *FUL* is all necessary for the lignification of cells in the en*b* layer (Ferrándiz et al., 2000; Liljegren et al., 2004).

In addition to *FUL*, the DZ-specific expression of *SHP1/2* and *IND* is also restricted by the *REPLUMLESS* (*RPL*), which encodes a homeodomain transcription factor and contributes to the specification of replum identity (Roeder et al., 2003). Expression of *SHP1/2* and *IND* is expanded into the replums in the *rpl* mutant genetic background (Figure 2; Roeder et al., 2003). Fruits from the *rpl* mutant are partially indehiscent due to loss of replum identity with ectopic cell lignification, in which the replum-lignified cells are coalesced into a single stripe that is connected with the lignified valve margin cells (Roeder et al., 2003). The loss of replum identity in *rpl* mutant can be largely rescued by further removal of *SHP1/2* activity, suggesting that the ectopic expression of *SHP1/2* is responsible for the *rpl* mutant phenotype (Roeder et al., 2003). Thus, both *RPL* and *FUL* are necessary for the proper development of a functional DZ by restricting the expression of *SHP1/2* in the valve margins. Recently, it was demonstrated that the *rpl* mutant phenotype can be rescued largely by *ap2* mutation (Ripoll et al., 2011). *AP2*, well known for its role in floral organ identity determination, encodes a transcription factor belonging to AP2/ERF family. *AP2* acts to prevent replum and valve margin overgrowth by negatively regulating replum and valve margin identity gene expression, respectively (Ripoll et al., 2011).

After the differentiation instruction of specific cell identity is established, the next step should be the final differentiation of distinct cell types. *NAC SECONDARY WALL THICKENING PROMOTING FACOTR1* (*NST1*) and *SECONDARY WALL-ASSOCIATED NAC DOMAIN PROTEIN1* (*SDN1*, also called *NST3*) are the master transcriptional switches controlling secondary cell wall thickening (Zhong et al., 2010). In the fruits, *NST1* and *SND1* are expressed in the valve en*b* layer, while only *NST1* is specifically expressed in the developing LL cells of DZs (Mitsuda and Ohme-Takagi, 2008). In the *nst1* null mutant, the fruits are indehiscent due to the loss of lignification of valve margin cells, and all the lignified cells except the vessel cells in the replums are lost in the *nst1 snd1* double mutant (Mitsuda and Ohme-Takagi, 2008). Expression of *SHP1/2* and *IND* appears to be normal in the *nst1 snd1* double mutant, suggesting that *NST1* and *SND1* act downstream of these transcription factors. Mitsuda and Ohme-Takagi (2008) further show that ectopic cell wall thickening in the valve cells in the *ful* mutant can be eliminated by mutation of *NST1*. Taken together, these data suggest *SHP1/2* regulate the lignification of valve margin cells by the path of *NST1* (Figure 2).

Intriguingly, *NST1* and *SND1* are predominantly expressed in the interfascicular fibers and xylems in the stems where *SHP1/2* are not expressed and are responsible for the secondary cell wall thickening in these cells (Zhong et al., 2006; Mitsuda et al., 2007). Furthermore, *NST1* and *SND1* are also identified as master regulators for xylem fiber differentiation (Zhong et al., 2006; Mitsuda et al., 2007; Oda and Fukuda, 2012). It is apparent that the developmental program of the stem interfascicular fibers and lignified valve margin cells are distinct. It seems that the valve margin specific expression of *NST1* represents an evolutionary innovation in the *cis*-regulatory elements that correlate with the establishment of lignified valve margin cells. How the *NST1* gene is co-opted in the *SHP1/2*-regulated network that direct the lignified valve margin cell development is an interesting question and worthy to be clarified in the future.

Prior to pod dehiscence, the cells in the separation layer secret enzymes to degrade the cell wall matrix, which bring about a reduction in cell-to-cell adhesion, thus facilitate the fruit to commit to dehiscence (Roberts et al., 2002). *ARABIDOPSIS DEHISCENCE ZONE POLYGALACTURONASE1* (*ADPG1*) and *ADPG2* encode plant specific endo-polygalacturonases (PGs) and are expressed in the separation layer of flower organs and fruit DZs (Ogawa et al., 2009). *ADPG1* and *ADPG2* are essential for enzymatic breakdown of cell middle lamella and are necessary for silique dehiscence, as genetic lesion in either genes leads to indehiscent fruits (Ogawa et al., 2009). *IND* is required for normal expression of *ADPG1* in the silique DZs (Ogawa et al., 2009). Thus, it seems that *ADPGs* are the final regulators of pod dehiscence in the separation layers (Figure 2).

### Hormonal Regulation of DZ Specification

Hormonal homeostasis and interactions are recently found as immediate downstream outputs from the core genetic network. Expression of *IND* is responsible for the formation of local auxin minimum in the valve margin by coordinating auxin efflux in the separation layer cells (Sorefan et al., 2009). Further analysis shows that another b-HLH transcription factor *SPATULA* (*SPT*), which is required for the carpel fusion early in female reproductive organ development, can interact with IND physically (Girin et al., 2011). The interaction of IND and SPT promotes the localization of PIN3 in the plasma membrane of valve margin cells to create the auxin depletion in the valve margin thus offering a proper hormonal environment for specific cell differentiation (Sorefan et al., 2009; Girin et al., 2011). Auxins and cytokinins often play an antagonistic role in plant development (Bishopp et al., 2011). Consistent with this scenario, the cytokinin signaling pathway is recently found to be active in the valve margin, and such a signaling pathway is disrupted in *shp1/2* and *ind* mutant. However, local application of cytokinin in developing fruits can restore valve margin formation and further increases dehiscence in *shp1/2* and *ind* mutants, suggesting that cytokinins play a crucial role in valve margin differentiation (Marsch-Martinez et al., 2012). In addition to auxins and cytokinins, gibberellins (GAs) have also recently been implicated as having roles in the establishment of separation layer cell identity (Arnaud et al., 2010). According to the “relief of restraint” model, GA-mediated degradation of DELLA protein is central to GA signaling and also required to activate downstream genes (Harberd, 2003; Sun and Gubler, 2004). GA3ox1, which catalyzes the final step in the synthesis of bioactive GAs, is demonstrated as the direct target of *IND*. ALC physically interacts with DELLA repressors, and the local production of GAs destabilize the DELLA protein and relieve the *ALC* to exert its function in SL cell specification (Arnaud et al., 2010). Taken together, these findings indicate involvement of several phytohormones in the specification of DZs and suggest that a precise balance between their biosynthesis and response is of fundamental importance. Notwithstanding with the investigations where the role of hormones in DZ development has been extensively explored, very few reports on how these hormonal signals are coordinated in the DZ are available. Therefore, one of the main challenges for future work remains to decipher the complete picture of the molecular mechanisms and interactions of plant hormones underlying DZ differentiation in dry fruits.

### Evolutionary Origin of Novel Fruit Characters by Modification of DZ Specification Genes

The family Brassicaceae contains over 300 genera, including a number of important vegetables and crops, such as broccoli and cauliflower (*Brassica oleracea*), oilseed rape (*Brassica napus*), and common radish (*Raphanus sativus*). As noted above, the basic fruit type in Brassicaceae is dry dehiscent silique, while there still exist bountiful morphological fruit variations within this family.

Heteroarthrocarpic fruit is a two-segmented fruit with an indehiscent distal part containing rudimentary ovules and a dehiscent proximal part consisting of normal ovules that develop into seeds. Phylogenetic reconstruction combined with morphological analysis shows that heteroarthrocarpic fruit has evolved multiple times within the tribe Brassiceae with nearly half of genera being heteroarthrocarpic (Hall et al., 2011). *Erucaria erucarioides* and *Cakile lanceolata* produce heteroarthrocarpic fruit with different dehiscent patterns. Avino et al. (2012) isolated the homologs of *SHP1/2*, *IND*, *ALC*, *FUL*, and *RPL* from both species and conducted comparative expression examinations. They found that the expression patterns of these genes in the fruit dehiscent segments are largely conserved between these species and in *Arabidopsis*, especially the genes that are involved in the establishment of valve margin identities (Avino et al., 2012). On the other hand, the fruit indehiscent segment is correlated with loss of gene expression of the entire valve margin genetic pathway. These expression data support the hypothesis that heteroarthrocarpy is evolved from dehiscent fruit via repositioning the valve margins (Avino et al., 2012).

Loss of fruit dehiscence has independently evolved in several genera across Brassicaceae (Appel and Al-Shehbaz, 2003). In the genus *Lepidium*, two phylogenetically related species, *L. campestre* and *L. appellianum*, bear dehiscent and indehiscent siliques, respectively (Mummenhoff et al., 2009). Mühlhausen et al. (2013) conducted a comparative analysis of the expression patterns of *SHP1/2*, *IND*, *ALC*, *FUL*, and *RPL* orthologs in these two species. They found that the expression patterns of these orthologous genes are highly conserved between *L. campestre* (dehiscent fruit) and *A. thaliana* (Mühlhausen et al., 2013). Transgenic plants of *L. campestr* with down-regulation of *SHP1/2*, *IND*, *ALC*, *FUL*, and *RPL* are found to be defective in fruit dehiscence; further anatomical examinations reveal that the fruit structure of these transgenic plants are similar to that of respective *Arabidopsis* mutant (Lenser and Theißen, 2013a). By contrast, the expression of these respective orthologs is completely abolished in the corresponding tissues of indehiscent *L. appellianum* fruit (Mühlhausen et al., 2013). These studies support the notion that the dehiscent network is basically conserved in Brassicaceae and further suggest that genetic changes in the upstream components of *SHP*-regulated pathway are responsible for the evolutionary origin of novel fruit characters (Mühlhausen et al., 2013). This idea is further supported from studies of Brassica species. *B. rapa* and *B. oleracea* produce dehiscent fruits and share similar anatomical structure with *A. thaliana* fruits. Functional analysis shows that *BraA.IND.a* and *BolC.IND.a* are orthologous to *IND* since mutation or down-regulation of either genes results in valve margin defect (Girin et al., 2010). Sequence alignment of the promoters of *IND*-like genes of *A. thaliana* and *B. rapa* reveals a 400-bp conserved sequence, which direct valve margin-specific expression of *IND* in *A. thaliana*. Further analysis shows that the specific activity of the 400-bp promoter sequence depends on the *SHP1/2* and *FUL* (Girin et al., 2010). An independent study in Brassica species reveals that loss of *RPL* gene expression is responsible for the evolutionary origin of the typical narrow replum in this genus. It is found that a point mutation in the promoter region significantly reduces *RPL* expression in the fruits and is associated with the narrow replum character (Arnaud et al., 2011). More recently, an independent research found that the genomic regions that encompass the key regulators of DZ specifying genes are associated with the natural variations in the pod dehiscence character in *Brassica napus* (Raman et al., 2014).

In *Medicago*, a genus of the legume family with a close phylogenetic relationship with Brassicaceae, some species develop coiled pods representing a novel strategy of collective seed dispersal. It is observed that the coiled pod morphology is tightly correlative with increased valve margin lignification, which is associated with a change in the protein sequence of *SHP* orthologs (Fourquin et al., 2013). Further analysis shows that the protein sequence modification alters the properties of the protein by affecting the affinity for other protein partners involved in a high-order complex (Fourquin et al., 2013). It is possible that *SHP*-directed secondary cell wall thickening is an evolutionary conserved module in Rosids (Ferrándiz and Fourquin, 2014). Nonetheless, it remains to determine the exact cellular and genetic basis that contributes to this indehiscent fruit morphology.

On the whole, the evidence outlined above points to a conserved genetic network controlling the pod dehiscence process and modifications of gene expression and protein properties in the core genetic components are associated with the origin of novel fruit characters.

## Regulation of Fruit Ripening and Shedding of Fleshy Fruits in Tomato

### Genetics of Fruit Ripening in Tomato

Like pod dehiscence in dry fruit species, the emergence of fleshy fruit represents another evolutionary innovation in which they attract animals for seed dispersal (Dilcher, 2000). Fleshy fruit can be divided into two classes, non-climacteric (e.g., strawberry and grape) and climacteric fruits (e.g., tomato and apple). In the fleshy model plant tomato (*Solanum lycopersicum*), the initiation of fruit ripening process is signified by a concomitant increase in respiration and biosynthesis of ethylene (Giovannoni, 2004; Seymour et al., 2008). In recent years, great advances have been made in dissecting the transcriptional regulation of ripening by the identification of genes with mutations that abolish the normal ripening process. Evidence shows that fruit ripening is a well-orchestrated process with the initiation of multiple genetic and biochemical pathways, which finally brings about the remarkable changes to the metabolic and physiological traits in a ripening fruit. The genetic regulation of the fruit ripening process has recently been thoroughly reviewed by several authors (Seymour et al., 2013; Ferrándiz and Fourquin, 2014). Here we only briefly introduce the genetic mechanisms underlying the fruit ripening process.

The *SEPALLATA4* clade of MADS-box gene *RIPENING INHIBITOR* (*RIN*) gene is demonstrated to act as the master switch of the fruit ripening process by directly activating the expression of *ACC Synthase 2* (*ACS2*), which is involved in the switch to system-2 ethylene production (Vrebalov et al., 2002; Martel et al., 2011). The spontaneous epigenetic modification of the promoter sequence of the SQUAMOSA Promoter Binding (SPB) protein encoded by the *COLORLESS NON-RIPENING* (*CNR*) gene decreases the expression level of *CNR* in the developing fruits, which effectively blocks the ripening process and results in fruits that fail to produce elevated ethylene at the onset of fruit ripening and an insensitivity to ethylene applications (Manning et al., 2006). Similar to the *rin* mutant, genetic lesions in the NAC transcription factor *NON-RIPENING* (*NOR*) gene lead to a non-ripening phenotype with a green fruit (Tigchelaar et al., 1973).

*TOMATO AGAMOUS LIKE1* (*TAGL1*), which encodes the orthologous gene of *AtSHP1/2*, is a positive regulator of fruit ripening (Itkin et al., 2009; Vrebalov et al., 2009). TAGL1 interacts with RIN to regulate the ethylene production by directly activating *ACS2* expression (Leseberg et al., 2008; Vrebalov et al., 2009). Overexpression of *TAGL1* in *Arabidopsis* results in an array of phenotypes that are similar to *SHP1/2* over-expressors, which points to a basically conserved role of *SHP*-like genes in organ identity determination (Pinyopich et al., 2003). However, the expression of *TAGL1* in *shp1/2* mutant genetic background only partially rescues the indehiscent fruit phenotype, indicating that *TAGL1* has evolved a novel function in fruit development compared with the *Arabidopsis* counterparts. Other positive regulators of fruit ripening include two closely related *FUL*-like homologs *FUL1* (also known as *TDR4*) and *FUL2* (also known as *MBP7*), which interact with RIN protein to regulate fruit ripening by coordinating the expression of genes involved in cell wall modification, cuticle production, volatile production, and glutamate accumulation (Bemer et al., 2012; Shima et al., 2013). Interestingly, the expression of *TAGL1* is found to be up-regulated in the pericarp of *FUL1/2* RNAi fruits, indicating a negative feedback loop from *FUL1/2* to *TAGL1* (Bemer et al., 2012). The negative regulation of *FUL* to *SHP* is also evident in the valve of *Arabidopsis* (Ferrándiz et al., 2000). These data point to a conservation of the regulatory network in the *FUL* and *SHP* between *Arabidopsis* and tomato. On the other hand, the homologs of the AP2-ERF protein SlAP2a are demonstrated to act as negative regulators of ripening by inhibiting ethylene biosynthesis and signaling pathways (Chung et al., 2010; Karlova et al., 2011). *SlAP2a* seems likely to act downstream of *CNR* as CNR protein can bind to the promoter of *SlAP2a in vitro* (Karlova et al., 2011).

As outlined above, it appears that genes (including *AP2*, *SHP*, and *FUL*) in fruit development are functionally conserved between *Arabidopsis* and tomato. In the case of the SHP-FUL module, it is plausible to assume that the genetic interaction between *SHP* and *FUL* in fruit development might have been established before the split of rosids and asterids. In the fleshy tomato, *SHP* and *FUL* are further co-opted in the *RIN*-regulated ethylene pathway to regulate fruit ripening subsequent upon sub-functionalization and neo-functionalization after lineage-specific gene duplication. The broad conservation of the *SHP*-*FUL* functional module in dry and fleshy fruits further suggest that fruit dehiscence and ripening may share a common origin and are parallel evolutionary innovations by recruiting a deeply conserved regulatory network (Ferrándiz and Fourquin, 2014).

### Genetic Control of Fruit Shedding in Tomato

**FIGURE 3:**
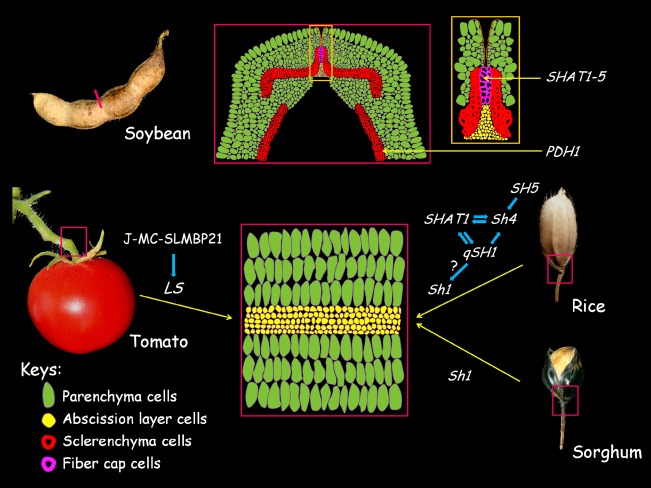
**Cellular basis of seed shattering in crops.** In soybeans, the loss of pod dehiscence is caused by the excessive lignification of the fiber cap cells (FCCs) and cell wall modification of the inner sclerenchyma cells in the pod valves (upper panel). The middle cartoon of the upper panel shows the transverse section of the pod ventral sutures with the yellow box shows the enlarged photo of the FCC and abscission layer. The loss of fruit shedding and seed shattering are due to the malfunction of the abscission zone (AZ) development of the pedicles (lower panel). The cartoon in the middle represents a transversal section of the AZ region as boxed in tomato, sorghum, and rice; genes with possible functional relationships that are involved in the AZ development in respective species are shown. The positive relationships between genes were shown by blue arrows. The figures and cartoons are not to scale.

In tomato (*Lycopersicon esculentum*), fruit shedding requires the proper development of the abscission zone (AZ) in the knuckle region of the pedicle (see reviews in Roberts et al., 2002; Estornell et al., 2013). The AZ is composed of several layers of smaller and densely cytoplasmic cells (Figure 3, lower panel). Cells in the AZ appear to be predetermined very early in development and are arrested in the following differentiation process (Roberts et al., 2002; Nocker, 2009). Several genes are found to be associated with the initial establishment and further differentiation of the AZ (Figure 3; also see reviews in Roberts et al., 2002). *JOINTLESS* (*J*), which encodes a *SVP/AGL24* clade MADS-box gene, is required for the proper AZ development, as *j* mutant fails to develop the AZ in the pedicle and fruit shedding does not occur normally (Mao et al., 2000). *MACROCALYX* (*MC*) encodes another MADS-box protein that falls into the AP1/FUL clade. Similar to the *j* mutant, the AZ is completely lost in the pedicle of *MC* RNAi plants (Nakano et al., 2012). Further analysis shows that the MC protein interacts physically with J to form a heterodimer with DNA-binding activity. It seems that *J* and *MC* regulate a common set of target genes, including transcription factors regulating meristem maintenance. These data further suggest the AZ cells possess meristematic potential (Roberts et al., 2002; Nakano et al., 2012). The J-MC protein complex has recently been extended to incorporate the SEP-like MADS-box protein SLMBP21. The SLMBP21 protein interacts with J and MC to form a higher-order protein complex to confer transactivation activity (Liu et al., 2014). Knockdown of *SLMBP21* completely abolishes AZ development, while overexpression of *SLMBP21* results in ectopic AZ-like cells at the proximal region of the pedicle (Liu et al., 2014). Because *J*, *MC*, and *SLMBP21* regulate a common set of target genes, it is possible that the obligate J-MC-SLMBP21 complex works synergistically to direct the expression of AZ development genes. In line with this notion, the expression of *LATERAL SUPPRESSOR (LS*), which encodes a VHID protein of the GARS transcription factor family, is found to be down-regulated in *j*, *mc*, and *SLMBP21* RNAi pedicles (Nakano et al., 2012; Liu et al., 2014). The *LS* was initially identified as a positive regulator of axillary meristem maintenance and the *ls* mutant also brings about impaired AZ development (Schumacher et al., 1999). It will be interesting to address how *LS* is co-opted in AZ cell meristematic potential maintenance under the control of J-MC-SLMBP21 complex-directed pathway.

## Convergent Evolution of the Non-Shattering Character in Domesticated Crops

From the evolutionary perspective, natural selection enables the wild plant species to possess elaborate mechanisms to disperse their seeds and fruits. While from the agronomic perspective, the natural seed dispersal is an undesired trait in crops as it leads to severe seed loss in harvest. As a result, natural seed dispersal is severely selected against by ancient humans to assure efficient cultivation during the domestication process (Harlan, 1992; Purugganan and Fuller, 2009; Lenser and Theißen, 2013b). The non-shattering or indehiscent character has been regarded as the milestone of domestication in the seed crops (such as cereals and legumes) as it renders the domesticated species more dependent on human activity for propagation and further facilitates the fixation of other domestication characters (Doebley et al., 2006; Purugganan and Fuller, 2009). In the seed crops, the reduction of seed shattering capability is evolved independently and is a convergent morphological adaptation to artificial selection (Doebley et al., 2006; Purugganan and Fuller, 2009; Lenser and Theißen, 2013b; Olsen and Wendel, 2013). In Section “Parallel Evolution of the Non-Shattering Trait in Cereal Crops,” we will review the cellular and genetic mechanisms underlying the morphological transition from shattering to non-shattering in domesticated crops (Figure 3, lower panel).

### Parallel Evolution of the Non-Shattering Trait in Cereal Crops

In cereal crops (such as rice and sorghum), the fruit dehiscence or seed shattering is implemented by an abscission layer in the joint between lemma and pedicel (Figure 3, lower panel). In rice (*Oryza sativa*), several transcription factor coding genes have been found to be associated with the reduction of seed shattering (Figure 3). *Shattering4* (*Sh4*) encodes a transcription factor with homology to Myb3 and is necessary for the development of a functional abscission layer in the pedicel (Li et al., 2006). A single amino acid change in the putative DNA biding domain is closely associated with the reduction in seed shattering in domesticated rice. In addition, the expression of the domesticated allele is also remarkably decreased compared with the wild allele (Li et al., 2006). Thus, it appears that the combination of coding and regulatory change of *Sh4* impairs the developmental program of the abscission layer, thus weakens the shattering phenotype (Li et al., 2006). *qSH1* is a major QTL on chromosome 1 controlling seed shattering in rice. The underlying gene, *qSH1*, encodes a BEL1-type homeobox transcription factor that is highly homologous to *AtRPL* (Konishi et al., 2006). *qSH1* is required for formation of the abscission layer in the pedicel. A single nucleotide polymorphism (SNP) in the 5′-regulatory region completely eliminates *qSH1* expression in the provisional abscission layer early in the development process and results in non-shattering trait in domesticated rice (Konishi et al., 2006). Notably, the regulatory SNP in the promoter sequence of *RPL* homologs is also responsible for the difference in seed dispersal structures produced by natural selection in *Brassica* species with reduced replum development (Arnaud et al., 2011). These examples demonstrate a remarkable convergent mechanism in which the same regulatory SNP can explain the developmental variations in seed dispersal structures relevant to both domestication and natural selection in a distantly related species (Arnaud et al., 2011; Gasser and Simon, 2011).

*SH5* is another BEL1-type homeobox gene with high homology to *qSH1*. *SH5* is highly expressed in the abscission layer (Yoon et al., 2014). Silencing of *SH5* suppresses the development of the abscission layer and inhibits seed shattering. Overexpression of *SH5* gives rise to an increase in seed shattering, a consequence of decreased lignin levels in the pedicel (Yoon et al., 2014). The expression of *Sh4* is found to be significantly up-regulated in the *SH5*-overexpressor, suggesting *SH5* positively regulates *Sh4* to direct abscission layer development (Yoon et al., 2014). Recently, the regulatory pathway of the abscission layer development was extended to include an AP2-transcription factor coding gene, *SHATTERING ABORTION1* (*SHAT1*, Zhou et al., 2012). *SHAT1* is required for seed shattering through specifying the abscission layer. The expression of *SHAT1* in the abscission layer is positively regulated by *Sh4*. *qSH1* expression is completely lost in the abscission layer in either *shat1* and *sh4* mutant background, suggesting *qSH1* functions downstream of *SHAT1* and *Sh4* in the establishment of the abscission layer (Zhou et al., 2012). Interestingly, *qSH1* is also required for the expression of *SHAT1* and *Sh4* in the abscission layer. Therefore, *qSH1* is probably involved in a positive feedback loop of *SHAT1* and *Sh4* by maintaining the expression of *SHAT1* and *Sh4* in the abscission layer (Zhou et al., 2012). Although *SH5* and *SHAT1* play roles in the differentiation of abscission layer, it remains to be determined whether these two genes are domestication genes targeted by artificial selection.

Similar to rice, the reducing of seed shattering in domesticated sorghum (*Sorghum bicolor*) results from the loss of abscission layer in the joint connecting the seed hull and pedicel. Seed shattering in sorghum is controlled by a single gene, *Shattering1* (*Sh1*), which encodes a YABBY transcription factor. The non-shattering character can be accounted for by one of three distinct loss-of-function mutations that are independently selected upon during the sorghum domestication process (Lin et al., 2012). Notably, the *Sh1* orthologs in rice and maize (*Zea mays*) harbor mutations that are possibly associated with the shattering reduction in respective crops (Paterson et al., 1995; Lin et al., 2012). Whether *Sh1* is rewired into the *SH5*-directed seed shattering network in rice remain to be explored in the future (Figure 3, lower panel). In *Sorghum propinquum*, a wild sorghum relative, seed shattering is conferred by the *SpWRKY* gene. It is postulated that *SpWRKY* negatively regulates cell wall biosynthesis genes in the abscission layer. Nonetheless, the *SpWRKY* has not been crafted by artificial selection to make a contribution to the non-shattering trait in domesticated sorghum (Tang et al., 2013). Taken together, these above findings have raised an intriguing possibility that the convergent domestication of non-shattering crops might have achieved through parallel selection on the same underlying genetic targets (Figure 3, lower panel; Lin et al., 2012; Lenser and Theißen, 2013b).

The *Q* gene in domesticated wheat (*Triticum aestivum*) is an important domestication gene as it confers the free-threshing character (the loss of tendency of the spike shattering; Simons et al., 2006). *Q* gene encodes a member of AP2-family transcription factor. The cultivated *Q* allele is transcribed more abundantly than the wild *q* allele. Furthermore, the two alleles also differ in a single amino acid that significantly enhances the homodimerization capacity of the domestication allele (Simons et al., 2006). Thus, similar to the case of *Sh4*, the evolution of the free-threshing trait in domesticated wheat may have attributed to the combination of both coding and regulatory changes in the domestication gene. The expression difference between *Q* and *q* seems more important as it can largely explain the free-threshing trait in the domesticated wheat (Simons et al., 2006; Zhang et al., 2011). Although the mutation that gives rise to *Q* had a profound effect in the domestication process of wheat as it enables the farmers to harvest the grain more efficiently, the exact cellular basis leading to the free-threshing trait is still unknown.

### The Domestication of Indehiscent Fruit in Legume Crops

In addition to cereals, loss of pod dehiscence also occurs in dicot crops, such as legumes. Species in the Legume family develop a characteristic dry dehiscent fruit (a legume or more generally a pod), which is derived from a monocarpellate pistil. The legume species disperse seeds by shattering the pod along the ventral suture after maturation (Tiwari and Bhatia, 1995). In cultivated soybean (*Glycine max*), the indehiscent pod is a major domestication trait that is targeted by artificial selection (Hymowitz, 1970; Harlan, 1992). The cellular basis and molecular mechanisms leading to the indehiscent pod have very recently been characterized. It is shown that the excessive lignification of the fiber cap cells (FCCs) in the ventral suture is responsible for the indehiscent fruit character (Figure 3, upper panel; Dong et al., 2014). Unexpectedly, the abscission layer is found to be functionally unchanged in the cultivated soybeans (Dong et al., 2014). *SHATTERING1–5* (*SHAT1–5*), which is homologous to *AtNST1/2* that acts as master transcriptional activator of secondary cell wall biosynthesis, resides in a QTL controlling pod dehiscence. Expression of *SHAT1*–5 is specifically localized in the developing FCCs. The lack of any fixed amino acid difference between the cultivated allele and wild allele, and that both alleles are capable of fully restoring the secondary cell wall thickening in the interfascicular fibers of *nst1-1;nst3-1* double mutant suggest that the differential expression of *SHAT1–5* in the FCC upon regulatory changes might be important for the indehiscent fruit. Using Laser Capture Microdissection system, Dong et al. (2014) reveal that a significant up-regulation of *SHAT1–5* in FCC of cultivated soybean is responsible for the excessive cell wall deposition in the FCC, which in turn prevents the pod from committing dehiscence after maturation (Figure 3, upper panel). Further analysis show that the over transcription of *SHAT1–5* in cultivated soybean FCC is attributable to the disruption of a repressive *cis*-regulatory element in the 5′-promoter region (Dong et al., 2014). Expression of *SHAT1–5* is related to the organs with severe secondary cell wall thickening, which is a common process during plant development (Dong et al., 2013). It seems that artificial selection would have discarded the null mutant in this gene due to pleiotropic effect, leaving a change in the specific regulatory element as a preferred mechanism for producing the desired phenotype.

*qPDH1* (QTL for Pod Dehiscence 1) is another major QTL controlling pod dehiscence in soybean that have very recently been cloned and shown to encode a dirigent-like protein with a possible function in lignin biosynthesis (Suzuki et al., 2010; Funatsuki et al., 2014). Expression of *PDH1* is correlated with the lignin deposition in the inner sclerenchyma of the pod walls (Figure 3, upper panel). *PDH1* promotes pod dehiscence by increasing the twisting force in the pod wall, which serves as a driving force for pod dehiscence (Funatsuki et al., 2014). In cultivated soybean, the indehiscent fruit is attributable to a premature stop codon in *PDH1*, which generates a non-functional protein (Funatsuki et al., 2014). Although the exact cellular and biochemical mechanisms leading to indehiscent pod by *PDH1* remain to be elucidated, it is apparent that artificial selection might have targeted multiple cellular mechanisms and the controlling genes, including *SHAT1–5* and *PDH1*, to minimize seed loss during soybean domestication. Meanwhile, these findings also raise an intriguing question as to how *SHAT1–5* and *PDH1* interact genetically to fine-tune the indehiscence degree of cultivated soybean that are adapted in different environments. Future analysis of allele frequency combined with careful phenotypic evaluation in a large collection of cultivated soybean germplasms would help to address this question.

The domesticated common bean (*Phaseolus valgaris*) originated in the Mesoamerican and Andean regions independently (Schmutz et al., 2014). Similar to other legume crops, the reduction of pod dehiscence represents a key domestication syndrome in the domesticated common bean. The indehiscent fruit results from the loss of fibers in the sutures (“stringless”), which is under the control of a major QTL, *St* locus (Koinange et al., 1996). *PvIND1*, a homolog of *AtIND* in common bean, was recently mapped in a region near the *St* locus. It appears that *PvIND* may not be directly involved in the control of pod dehiscence and may not be the causal gene underlying *St*, as polymorphism in the *PvIND* gene fails to link with the genotype on *St* locus and co-segregate with the dehiscent/indehiscent phenotype (Gioia et al., 2013). While *PvIND* is postulated as the *AtIND* homolog based on sequence homology in the conserved b-HLH domain, the *IND*-related transcription factors are specific to Brassicaceae and its role in valve margin cell lignification may have been acquired since the duplication event happened recently in the *HECATE3* (*HEC3*) gene clade in Brassicaceae (Liljegren et al., 2004; Girin et al., 2010). Therefore, it is possible that polymorphisms in other *AtIND* homologs in the common bean genome may have been associated with pod indehiscence. Alternatively, considering that the fibers are mainly composed of sclerenchyma cells with well-developed secondary cell walls, it is also likely that genes involved in the regulation of secondary cell wall deposition or fiber cell differentiation may have contributed to the *St* locus in controlling pod dehiscence. Future work is necessary to discriminate these possibilities.

## Conclusions and Future Perspectives

In the past 15 years, our understanding of the genetic control and evolution of the seed shattering/pod dehiscence processes has been advanced significantly by the implement of a combination of multiple experimental approaches. In *Arabidopsis*, the homostasis and interaction of hormones is revealed to work in another regulation layer in establishing the DZs. The core regulatory module (*SHP*-*FUL*) controlling DZ development is found to be largely conserved in dry fruit species that are closely relative to *Arabidopsis* while modification of the key regulatory genes frequently contributes to the evolution of specialized fruit morphology with novel dispersal strategies. Studies in the genetic control of the fleshy fruit maturation process further extend the conservation of *SHP*-*FUL* module into angiosperms and suggest that fruit dehiscence and ripening are in parallel evolved characters by co-opting the same underlying regulatory networks. Although we can now begin to understand the molecular and biochemical basis of fruit dehiscence and ripening in model species, a challenge remains to obtain greater molecular data from other non-model species, to unveil the evolutionary mechanisms of fruit diversification widespread in nature.

In the domesticated crops, it is apparent that the convergent evolution of non-shattering (indehiscent) fruit is often employed by the same gene or strikingly, the same mutation, while non-homologous genes are also frequently evident in different crops. In the future, with the growing interest in the molecular mechanisms of domesticated syndromes that arise as the result of evolutionary implications and their agriculture importance, an equally important and complementary issue will be the advances in the application of high throughput sequencing technology (next-generation sequencing, NGS) combined with genotype-phenotype associations (genome-wide association analysis, GWAS) to zoom in on the exact mutations leading to the non-shattering character in additional crops. Overall, the list of genes that participate in the seed shattering process has experienced an unprecedented explosion in the past few years (Table 1), we can now begin to think about how to translate this basic knowledge into practice in crop breeding programs to feed the world in the face of growing population pressures. Exemplary work has been done in *Brassica juncea* by over-expression *AtFUL* to make pods resistant to shattering (Østergaard et al., 2006).

**TABLE 1:** **Summary of genes involved in the seed shattering process**.

**Species**	**Gene(s)**	**Gene category**	**Molecular function**	**Phenotypic effect**	**References**
*Arabidopsis thaliana*	*SHATTERPROOF1/2*	Transcription factor	Transcriptional regulator (MADS)	Indehiscent pod	Liljegren et al. (2000)
	*INDEHISCENT*	Transcription factor	Transcriptional regulator (bHLH)	Indehiscent pod	Liljegren et al. (2004)
	*ALCATRAZ*	Transcription factor	Transcriptional regulator (bHLH)	Partially indehiscent pod	Rajani and Sundaresan (2001)
	*FRUITFULL*	Transcription factor	Transcriptional regulator (MADS)	Premature bursting pod	Gu et al. (1998)
	*REPLUMLESS*	Transcription factor	Transcriptional regulator (homeodomain)	Partially indehiscent pod	Roeder et al. (2003)
	*NST1/3*	Transcription factor	Transcriptional regulator (NAC)	Indehiscent pod	Mitsuda and Ohme-Takagi (2008)
	*ADPG1/2*	Endo-polygalacturonase	Degrade cell wall matrix	Indehiscent pod	Ogawa et al. (2009)
	*GA3ox1*	Catalytic enzyme	GA biosynthesis	Partially indehiscent pod	Arnaud et al. (2010)
*Glycine max*	*SHATTERING1–5*	Transcription factor	Transcriptional regulator (NAC)	Indehiscent pod	Dong et al. (2014)
	*PDH1*	Dirigent-like protein	Lignin biosynthesis	Indehiscent pod	Funatsuki et al. (2014)
*Solanum lycopersicum*	*JOINTLESS*	Transcription factor	Transcriptional regulator (MADS)	Non-shedding fruit	Mao et al. (2000)
	*MACROCALYX*	Transcription factor	Transcriptional regulator (MADS)	Non-shedding fruit	Nakano et al. (2012)
	*SLMBP21*	Transcription factor	Transcriptional regulator (MADS)	Non-shedding fruit	Liu et al. (2014)
	*LATERAL SUPPRESSOR*	Transcription factor	Transcriptional regulator (GARS)	Non-shedding fruit	Schumacher et al. (1999)
*Oryza sativa*	*Shattering4*	Transcription factor	Transcriptional regulator (Myb)	Non-shattering seed	Li et al. (2006)
	*qSH1*	Transcription factor	Transcriptional regulator (homeodomain)	Non-shattering seed	Konishi et al. (2006)
	*SH5*	Transcription factor	Transcriptional regulator (homeodomain)	Non-shattering seed	Yoon et al. (2014)
	*SHATTERING ABORTION1*	Transcription factor	Transcriptional regulator (AP2)	Non-shattering seed	Zhou et al. (2012)
	*Shattering1*	Transcription factor	Transcriptional regulator (YABBY)	Non-shattering seed?	Lin et al. (2012)
*Sorghum bicolor*	*Shattering1*	Transcription factor	Transcriptional regulator (YABBY)	Non-shattering seed	Lin et al. (2012)
*Sorghum propinquum*	*SpWRKY*	Transcription factor	Transcriptional regulator (WRKY)	Non-shattering seed	Tang et al. (2013)
*Zea mays*	*Shattering1*	Transcription factor	Transcriptional regulator (YABBY)	Non-shattering seed?	Lin et al. (2012)
*Triticum aestivum*	*Q*	Transcription factor	Transcriptional regulator (AP2/ERF)	Free-threshing character	Simons et al. (2006), Zhang et al. (2011)

### Conflict of Interest Statement

The authors declare that the research was conducted in the absence of any commercial or financial relationships that could be construed as a potential conflict of interest.

## References

[B1] Alonso-Cantabrana H. Ripoll J. J. Ochando I. Vera A. Ferrándiz C. Martínez-Laborda A. (2007). Common regulatory networks in leaf and fruit patterning revealed by mutations in the *Arabidopsis* ASYMMETRIC LEAVES1 gene. Development 134, 2663–2671. 10.1242/dev.02864 17592013

[B2] Appel O. Al-Shehbaz I. A. (2003). Cruciferae. Flowering Plants–Dicotyledons. pp. 75–174. Berlin: Springer.

[B3] Arnaud N. Girin T. Sorefan K. Fuentes S. Wood T. A. Lawrenson T. (2010). Gibberellins control fruit patterning in *Arabidopsis thaliana*. Genes Dev. 24, 2127–2132. 10.1101/gad.593410 20889713 PMC2947765

[B4] Arnaud N. Lawrenson T. Østergaard L. Sablowski R. (2011). The same regulatory point mutation changed seed-dispersal structures in evolution and domestication. Curr. Biol. 21, 1215–1219. 10.1016/j.cub.2011.06.008 21737279

[B5] Avino M. Kramer E. M. Donohuem K. Hammel A. J. Hall J. C. (2012). Understanding the basis of a novel fruit type in Brassicaceae-conservation and deviation in expression patterns of six genes. Evodevo 3, 20. 10.1186/2041-9139-3-20 22943452 PMC3503883

[B6] Bemer M. Karlova R. Ballester A. R. Tikunov Y. M. Bovy A. G. Wolters-Arts M. (2012). The tomato FRUITFULL homologs TDR4/FUL1 and MBP7/FUL2 regulate ethylene-independent aspects of fruit ripening. Plant Cell 24, 4437–4451. 10.1105/tpc.112.103283 23136376 PMC3531844

[B7] Bishopp A. Benkova E. Helariutta Y. (2011). Sending mixed messages: auxin–cytokinin crosstalk in roots. Curr. Opin. Plant Biol. 14, 10–16. 10.1016/j.pbi.2010.08.014 20926335

[B8] Chung M. Y. Vrebalov J. Alba R. Lee J. McQuinn R. Chung J. D. (2010). A tomato (*Solanum lycopersicum*) APETALA2/ERF gene, SlAP2a, is a negative regulator of fruit ripening. Plant J. 64, 936–947. 10.1111/j.1365-313X.2010.04384.x 21143675

[B9] Colombo M. Brambilla V. Marcheselli R. Caporali E. Kater M. M. Colombo L. (2009). A new role for the SHATTERPROOF genes during *Arabidopsis* gynoecium development. Dev. Biol. 337, 294–302. 10.1016/j.ydbio.2009.10.043 19900437

[B10] Dilcher D. (2000). Toward a new synthesis major evolutionary trends in the angiosperm fossil record. Proc. Natl. Acad. Sci. U.S.A. 97, 7030–7036. 10.1073/pnas.97.13.7030 10860967 PMC34380

[B11] Dinneny J. R. Weigel D. Yanofsky M. F. (2005). A genetic framework for fruit patterning in *Arabidopsis thaliana*. Development 132, 4687–4696. 10.1242/dev.02062 16192305

[B12] Doebley J. F. Gaut B. S. Smith B. D. (2006). The molecular genetics of crop domestication. Cell 127, 1309–1321. 10.1016/j.cell.2006.12.006 17190597

[B13] Dong Y. Wang B. H. Wang Y. Z. (2013). Functional characterization of the orthologs of AtNST1/2 in *Glycine soja* (Fabaceae) and the evolutionary implications. J. Syst. Evol. 51, 693–703. 10.1111/jse.12025

[B14] Dong Y. Yang X. Liu J. Wang B. H. Liu B. L. Wang Y. Z. (2014). Pod dehiscence resistance associated with domestication is mediated by a NAC gene in soybean. Nat. Commun. 5, 3352. 10.1038/ncomms4352 24549030

[B15] Estornell L. H. Agusti J. Merelo P. Talon M. Tadeo F. R. (2013). Elucidating mechanisms underlying organ abscission. Plant Sci. 199–200, 48–60. 10.1016/j.plantsci.2012.10.008 23265318

[B16] Ferrándiz C. (2002). Regulation of fruit dehiscence in *Arabidopsis*. J. Exp. Bot. 53, 2031–2038. 10.1093/jxb/erf082 12324527

[B17] Ferrándiz C. Fourquin C. (2014). Role of the FUL-SHP network in the evolution of fruit morphology and function. J. Exp. Bot. 65, 4505–4513. 10.1093/jxb/ert479 24482369

[B18] Ferrándiz C. Liljegren S. J. Yanofsky M. F. (2000). Negative regulation of the SHATTERPROOF genes by FRUITFULL during *Arabidopsis* fruit development. Science 289, 436–438. 10.1126/science.289.5478.436 10903201

[B19] Ferrándiz C. Pelaz S. Yanofsky M. F. (1999). Control of carpel and fruit development in *Arabidopsis*. Annu. Rev. Biochem. 68, 321–354. 10.1146/annurev.biochem.68.1.321 10872453

[B20] Fourquin C. Cerro C. Victoria F. C. Vialette-Guiraud A. Oliveira A. C. Ferrandiz C. (2013). A change in SHATTERPROOF protein lies at the origin of a fruit morphological novelty and a new strategy for seed dispersal in *Medicago* genus. Plant Physiol. 162, 907–917. 10.1104/pp.113.217570 23640757 PMC3668079

[B21] Funatsuki H. Suzuki M. Hirose A. Inaba H. Yamada T. Hajika M. (2014). Molecular basis of a shattering resistance boosting global dissemination of soybean. Proc. Natl. Acad. Sci. U.S.A. 111, 17797–17802. 10.1073/pnas.1417282111 25468966 PMC4273335

[B22] Gasser C. S. Simon M. K. (2011). Seed dispersal: same gene, different organs. Curr. Biol. 21, R546–R548. 10.1016/j.cub.2011.06.039 21783033

[B23] Gioia T. Logozzo G. Kami J. Spagnoletti Zeuli P. Gepts P. (2013). Identification and characterization of a homologue to the *Arabidopsis* INDEHISCENT gene in common bean. J. Hered. 104, 273–286. 10.1093/jhered/ess102 23235700

[B24] Giovannoni J. J. (2004). Genetic regulation of fruit development and ripening. Plant Cell 16, S170–S180. 10.1105/tpc.019158 15010516 PMC2643394

[B25] Girin T. Paicu T. Stephenson P. Fuentes S. Korner E. O’Brien M. (2011). INDEHISCENT and SPATULA interact to specify carpel and valve margin tissue and thus promote seed dispersal in *Arabidopsis*. Plant Cell 23, 3641–3653. 10.1105/tpc.111.090944 21990939 PMC3229140

[B26] Girin T. Stephenson P. Goldsack C. M. Kempin S. A. Perez A. Pires N. (2010). Brassicaceae INDEHISCENT genes specify valve margin cell fate and repress replum formation. Plant J. 63, 329–338. 10.1111/j.1365-313X.2010.04244.x 20444234

[B27] Gu Q. Ferrándiz C. Yanofsky M. F. Martienssen R. (1998). The FRUITFULL MADS-box gene mediates cell differentiation during *Arabidopsis* fruit development. Development 125, 1509–1517. 9502732 10.1242/dev.125.8.1509

[B28] Hall J. C. Tisdale T. E. Donohue K. Wheeler A. Al-Yahya M. A. Kramer E. M. (2011). Convergent evolution of a complex fruit structure in the tribe Brassiceae (Brassicaceae). Am. J. Bot. 98, 1989–2003. 10.3732/ajb.1100203 22081414

[B29] Harlan J. R. (1992). Crops and Man. Madison, WI: American Society of Agronomy.

[B30] Harberd N. P. (2003). Relieving DELLA restraint. Science 299, 1853–1854. 10.1126/science.1083217 12649470

[B31] Hay A. Barkoulas M. Tsiantis M. (2006). ASYMMETRIC LEAVES1 and auxin activities converge to repress BREVIPEDICELLUS expression and promote leaf development in *Arabidopsis*. Development 133, 3955–3961. 10.1242/dev.02545 16971475

[B32] Hymowitz T. (1970). On the domestication of soybeans. Econ. Bot. 24, 408–421.

[B33] Itkin M. Seybold H. Breitel D. Rogachev I. Meir S. Aharoni A. (2009). TOMATO AGAMOUS-LIKE 1 is a component of the fruit ripening regulatory network. Plant J. 60, 1081–1095. 10.1111/j.1365-313X.2009.04064.x 19891701

[B34] Karlova R. Rosin F. M. Busscher-Lange J. Parapunova V. Do P. T. Fernie A. R. (2011). Transcriptome and metabolite profiling show that APETALA2a is a major regulator of tomato fruit ripening. Plant Cell 23, 923–941. 10.1105/tpc.110.081273 21398570 PMC3082273

[B35] Koinange E. M. Singh S. P. Gepts P. (1996). Genetic control of the domestication syndrome in common bean. Crop Sci. 36, 1037–1045. 10.2135/cropsci1996.0011183X003600040037x

[B36] Konishi S. Izawa T. Lin S. Y. Ebana K. Fukuta Y. Sasaki T. (2006). An SNP caused loss of seed shattering during rice domestication. Science 312, 1392–1396. 10.1126/science.1126410 16614172

[B37] Lenser T. Theißen G. (2013a). Conservation of fruit dehiscence pathways between *Lepidium campestre* and *Arabidopsis thaliana* sheds light on the regulation of INDEHISCENT. Plant J. 76, 545–556. 10.1111/tpj.12321 24004048

[B38] Lenser T. Theißen G. (2013b). Molecular mechanisms involved in convergent crop domestication. Trends Plant Sci. 18, 704–714. 10.1016/j.tplants.2013.08.007 24035234

[B39] Leseberg C. H. Eissler C. L. Wang X. Johns M. A. Duvall M. R. Mao L. (2008). Interaction study of MADS-domain proteins in tomato. J. Exp. Bot. 59, 2253–2265. 10.1093/jxb/ern094 18487636

[B40] Lewis M. W. Leslie M. E. Liljegren S. J. (2006). Plant separation: 50 ways to leave your mother. Curr. Opin. Plant Biol. 9, 59–65. 10.1016/j.pbi.2005.11.009 16337172

[B41] Li C. Zhou A. Sang T. (2006). Rice domestication by reducing shattering. Science 311, 1936–1939. 10.1126/science.1123604 16527928

[B42] Liljegren S. J. Ditta G. S. Eshed Y. Savidge B. Bowman J. Yanofsky M. F. (2000). SHATTERPROOF MADS-box genes control seed dispersal in *Arabidopsis*. Nature 404, 766–770. 10.1038/35008089 10783890

[B43] Liljegren S. J. Roeder A. H. Kempin S. A. Gremski K. Østergaard L. Guimil S. (2004). Control of fruit patterning in *Arabidopsis* by INDEHISCENT. Cell 116, 843–853. 10.1016/S0092-8674(04)00217-X 15035986

[B44] Lin Z. Li X. Shannon L. M. Yeh C. T. Wang M. L. Bai G. (2012). Parallel domestication of the Shattering1 genes in cereals. Nat. Genet. 44, 720–724. 10.1038/ng.2281 22581231 PMC3532051

[B45] Liu D. Wang D. Qin Z. Zhang D. Yin L. Wu L. (2014). The SEPALLATA MADS-box protein SLMBP21 forms protein complexes with JOINTLESS and MACROCALYX as a transcription activator for development of the tomato flower abscission zone. Plant J. 77, 284–296. 10.1111/tpj.12387 24274099

[B46] Manning K. Tor M. Poole M. Hong Y. Thompson A. J. King G. J. (2006). A naturally occurring epigenetic mutation in a gene encoding an SBP-box transcription factor inhibits tomato fruit ripening. Nat. Genet. 38, 948–952. 10.1038/ng1841 16832354

[B47] Mao L. Begum D. Chuang H. Budlman M. A. Szymkowiak E. J. Irish E. E. (2000). JOINTLESS is a MADS-box gene controlling tomato flower abscission zone development. Nature 406, 910–913. 10.1038/35022611 10972295

[B48] Marsch-Martinez N. Ramos-Cruz D. Reyes-Olalde J. Lozano-Sotomayor P. Zuniga-Mayo V. M. Folter S. (2012). The role of cytokinin during *Arabidopsis* gynoecia and fruit morphogenesis and patterning. Plant J. 72, 222–234. 10.1111/j.1365-313X.2012.05062.x 22640521

[B49] Martel C. Vrebalov J. Tafelmeyer P. Giovannoni J. J. (2011). The tomato MADS-box transcription factor RIPENING INHIBITOR interacts with promoters involved in numerous ripening processes in a COLORLESS NONRIPENING-dependent manner. Plant Physiol. 157, 1568–1579. 10.1104/pp.111.181107 21941001 PMC3252172

[B50] Mitsuda N. Iwase A. Yamamoto H. Yoshida M. Seki M. Shinozaki K. (2007). NAC transcription factors, NST1 and NST3, are key regulators of the formation of secondary walls in woody tissues of *Arabidopsis*. Plant Cell 19, 270–280. 10.1105/tpc.106.047043 17237351 PMC1820955

[B51] Mitsuda N. Ohme-Takagi M. (2008). NAC transcription factors NST1 and NST3 regulate pod dehiscence in a partially redundant manner by promoting secondary wall formation after the establishment of tissue identity. Plant J. 56, 768–778. 10.1111/j.1365-313X.2008.03633.x 18657234

[B52] Mühlhausen A. Lenser T. Mummenhoff K. Theißen G. (2013). Evidence that an evolutionary transition from dehiscent to indehiscent fruits in *Lepidium* (Brassicaceae) was caused by a change in the control of valve margin identity genes. Plant J. 73, 824–835. 10.1111/tpj.12079 23173897

[B53] Mummenhoff K. Polster A. Mühlhausen A. Theißen G. (2009). Lepidium as a model system for studying the evolution of fruit development in Brassicaceae. J. Exp. Bot. 60, 1503–1513. 10.1093/jxb/ern304 19052256

[B54] Nakano T. Kimbara J. Fujisawa M. Kitagawa M. Ihashi N. Maeda H. (2012). MACROCALYX and JOINTLESS interact in the transcriptional regulation of tomato fruit abscission zone development. Plant Physiol. 158, 439–450. 10.1104/pp.111.183731 22106095 PMC3252084

[B55] Nathan R. Muller-Landau H. C. (2000). Spatial patterns of seed dispersal, their determinants and consequences for recruitment. Trends Ecol. Evol. 15, 278–285. 10.1016/S0169-5347(00)01874-7 10856948

[B56] Nocker S. (2009). Development of the abscission zone. Stewart Postharvest. Rev. 5, 1–6. 10.2212/spr.2009.1.5

[B57] Oda Y. Fukuda H. (2012). Secondary cell wall patterning during xylem differentiation. Curr. Opin. Plant Biol. 15, 38–44. 10.1016/j.pbi.2011.10.005 22078063

[B58] Ogawa M. Kay P. Wilson S. Swain S. M. (2009). ARABIDOPSIS DEHISCENCE ZONE POLYGALACTURONASE1 (ADPG1), ADPG2, and QUARTET2 are Polygalacturonases required for cell separation during reproductive development in *Arabidopsis*. Plant Cell 21, 216–233. 10.1105/tpc.108.063768 19168715 PMC2648098

[B59] Olsen K. M. Wendel J. F. (2013). A bountiful harvest: genomic insights into crop domestication phenotypes. Annu. Rev. Plant Biol. 64, 47–70. 10.1146/annurev-arplant-050312-120048 23451788

[B60] Østergaard L. (2009). Don’t ‘leaf’ now. The making of a fruit. Curr. Opin. Plant Biol. 12, 36–41. 10.1016/j.pbi.2008.09.011 19013099

[B61] Østergaard L. Kempin S. A. Bies D. Klee H. J. Yanofsky M. F. (2006). Pod shatter-resistant Brassica fruit produced by ectopic expression of the FRUIT-FULL gene. Plant Biotechnol. J. 4, 45–51. 10.1111/j.1467-7652.2005.00156.x 17177784

[B62] Pinyopich A. Ditta G. S. Savidge B. Liljegren S. J. Baumann E. Wisman E. (2003). Assessing the redundancy of MADS-box genes during carpel and ovule development. Nature 424, 85–88. 10.1038/nature01741 12840762

[B63] Paterson A. H. Lin Y. Li Z. Schertz K. F. Doebley J. F. Pinson S. R. M. (1995). Convergent domestication of cereal crops by independent mutations at corresponding genetic loci. Science 269, 1714–1718. 10.1126/science.269.5231.1714 17821643

[B64] Purugganan M. D. Fuller D. Q. (2009). The nature of selection during plant domestication. Nature 457, 843–848. 10.1038/nature07895 19212403

[B65] Rajani S. Sundaresan V. (2001). The *Arabidopsis* myc-bHLH gene ALCATRAZ enables cell separation in fruit dehiscence. Curr. Biol. 11, 1914–1922. 10.1016/S0960-9822(01)00593-0 11747817

[B66] Raman H. Raman R. Kilian A. Detering F. Carling J. Coombes N. (2014). Genome-wide delineation of natural variation for pod shatter resistance in *Brassica napus*. PLoS ONE 9:e101673. 10.1371/journal.pone.0101673 25006804 PMC4090071

[B67] Ripoll J. J. Roeder A. H. Ditta G. S. Yanofsky M. F. (2011). A novel role for the floral homeotic gene APETALA2 during *Arabidopsis* fruit development. Development 138, 5167–5176. 10.1242/dev.073031 22031547

[B68] Roberts J. A. Elliott K. A. Gonzalez-Carranza Z. H. (2002). Abscission, dehiscence, and other cell separation processes. Annu. Rev. Plant Biol. 53, 131–158. 10.1146/annurev.arplant.53.092701.180236 12221970

[B69] Roeder A. H. K. Ferrándiz C. Yanofsky M. F. (2003). The role of the REPLUMLESS homeodomain protein in patterning the *Arabidopsis* fruit. Curr. Biol. 13, 1630–1635. 10.1016/j.cub.2003.08.027 13678595

[B70] Schmutz J. McClean P. E. Mamidi S. Wu G. A. Cannon S. B. Grimwood J. (2014). A reference genome for common bean and genome-wide analysis of dual domestications. Nat. Genet. 46, 707–713. 10.1038/ng.3008 24908249 PMC7048698

[B71] Schumacher K. Schmitt T. Rossberg M. Schmitz G. Theres K. (1999). The lateral suppressor (Ls) gene of tomato encodes a new member of the VHIID protein family. Proc. Natl. Acad. Sci. U.S.A. 96, 290–295. 9874811 10.1073/pnas.96.1.290 PMC15132

[B72] Seymour G. Poole M. Manning K. King G. J. (2008). Genetics and epigenetics of fruit development and ripening. Curr. Opin. Plant Biol. 11, 58–63. 10.1016/j.pbi.2007.09.003 17997126

[B73] Seymour G. B. Østergaard L. Chapman N. H. Knapp S. Martin C. (2013). Fruit development and ripening. Annu. Rev. Plant Biol. 64, 219–241. 10.1146/annurev-arplant-050312-120057 23394500

[B74] Shima Y. Kitagawa M. Fujisawa M. Nakano T. Kato H. Kimbara J. (2013). Tomato FRUITFULL homologues act in fruit ripening via forming MADS-box transcription factor complexes with RIN. Plant Mol. Biol. 82, 427–438. 10.1007/s11103-013-0071-y 23677393

[B75] Simons K. J. Fellers J. P. Trick H. N. Zhang Z. Tai Y. S. Gill B. S. (2006). Molecular characterization of the major wheat domestication gene Q. Genetics 172, 547–555. 10.1534/genetics.105.044727 16172507 PMC1456182

[B76] Sorefan K. Girin T. Liljegren S. J. Ljung K. Robles P. Galvan-Ampudia C. S. (2009). A regulated auxin minimum is required for seed dispersal in *Arabidopsis*. Nature 459, 583–586. 10.1038/nature07875 19478783

[B77] Sun T. P. Gubler F. (2004). Molecular mechanism of gibberellin signaling in plants. Annu. Rev. Plant Biol. 55, 197–223. 10.1146/annurev.arplant.55.031903.141753 15377219

[B78] Suzuki M. Fujino K. Nakamoto Y. Ishimoto M. Funatsuki H. (2010). Fine mapping and development of DNA markers for the qPDH1 locus associated with pod dehiscence in soybean. Mol. Breed. 25, 407–418. 10.1007/s11032-009-9340-5

[B79] Tang H. Cuevas H. E. Das S. Sezen U. U. Zhou C. Guo H. (2013). Seed shattering in a wild sorghum is conferred by a locus unrelated to domestication. Proc. Natl. Acad. Sci. U.S.A. 110, 15824–15829. 10.1073/pnas.1305213110 24019506 PMC3785776

[B80] Tigchelaar E. C. Tomes M. L. Kerr E. A. Barman R. J. (1973). A new fruit ripening mutant, non-ripening (nor). Rep Tomato Genet. Coop. 23, 33.

[B81] Tiwari S. P. Bhatia V. S. (1995). Character of pod anatomy associated with resistance to pod dehiscence in soybean. Ann. Bot. 76, 483–485. 10.1006/anbo.1995.1123

[B82] Vrebalov J. Pan I. L. Arroyo A. J. McQuinn R. Chung M. Poole M. (2009). Fleshy fruit expansion and ripening are regulated by the tomato SHATTERPROOF gene TAGL1. Plant Cell 21, 3041–3062. 10.1105/tpc.109.066936 19880793 PMC2782289

[B83] Vrebalov J. Ruezinsky D. Padmanabhan V. White R. Medrano D. Drake R. (2002). A MADS-box gene necessary for fruit ripening at the tomato ripening inhibitor (rin) locus. Science 296, 343–346. 10.1126/science.1068181 11951045

[B84] Yoon J. Cho L. H. Kim S. L. Choi H. Koh H. J. An G. (2014). The BEL1-type homeobox gene SH5 induces seed shattering by enhancing abscission-zone development and inhibiting lignin biosynthesis. Plant J. 79, 717–728. 10.1111/tpj.12581 24923192

[B85] Zhang Z. Belcram H. Gornicki P. Charles M. Just J. Huneau C. (2011). Duplication and partitioning in evolution and function of homoeologous Q loci governing domestication characters in polyploid wheat. Proc. Natl. Acad. Sci. U.S.A. 108, 18737–18742. 10.1073/pnas.1110552108 22042872 PMC3219148

[B86] Zhong R. Demura T. Ye Z. H. (2006). SND1, a NAC domain transcription factor, is a key regulator of secondary wall synthesis in fibers of *Arabidopsis*. Plant Cell 18, 3158–3170. 10.1105/tpc.106.047399 17114348 PMC1693950

[B87] Zhong R. Lee C. Ye Z. H. (2010). Evolutionary conservation of the transcriptional network regulating secondary cell wall biosynthesis. Trends Plant Sci. 15, 625–632. 10.1016/j.tplants.2010.08.007 20833576

[B88] Zhou Y. Lu D. Li C. Luo J. Zhu B. F. Zhu J. (2012). Genetic control of seed shattering in rice by the APETALA2 transcription factor shattering abortion1. Plant Cell 24, 1034–1048. 10.1105/tpc.111.094383 22408071 PMC3336138

